# Antihypertensive Medication and Dementia Risk in Older Adult African Americans with Hypertension: A Prospective Cohort Study

**DOI:** 10.1007/s11606-017-4281-x

**Published:** 2018-01-12

**Authors:** Michael D. Murray, Hugh C. Hendrie, Kathleen A. Lane, Mengjie Zheng, Roberta Ambuehl, Shanshan Li, Frederick W. Unverzagt, Christopher M. Callahan, Sujuan Gao

**Affiliations:** 1 Regenstrief Institute, Inc., Indianapolis, IN USA; 2 Purdue University College of Pharmacy, West Lafayette, IN USA; 3 Indiana University Center for Aging Research, Indianapolis, IN USA; 4 Department of Psychiatry, Indiana University School of Medicine, Indianapolis, IN USA; 5 Department of Biostatistics, Indiana University School of Medicine, Indianapolis, IN USA; 6 Division of Internal Medicine and Geriatrics, Department of Medicine, Indiana University School of Medicine, Indianapolis, IN USA

**Keywords:** African Americans, antihypertensives, dementia, hypertension, prospective cohort

## Abstract

**Background:**

African Americans are especially at risk of hypertension and dementia. Antihypertensive medications reduce the risk of cardiovascular events, but may also reduce the risk of dementia.

**Objective:**

To assess the longitudinal effects of antihypertensive medications and blood pressure on the onset of incident dementia in a cohort of African Americans.

**Design:**

Prospective cohort.

**Participants:**

1236 community-dwelling patients from an inner-city public health care system, aged 65 years and older, with a history of hypertension but no history of dementia, and who had at least three primary care visits and a prescription filled for any medication.

**Main Measures:**

Blood pressure was the average of three seated measurements. Dementia was diagnosed using a two-stage design, with a screening evaluation every 2 to 3 years followed by a comprehensive in-home clinical evaluation for those with a positive screen. Laboratory, inpatient and outpatient encounter data, coded diagnoses and procedures, and medication records were derived from a health information exchange.

**Key Results:**

Of the 1236 hypertensive participants without dementia at baseline, 114 (9%) developed incident dementia during follow-up. Individuals prescribed any antihypertensive medication (*n* = 816) were found to have a significantly reduced risk of dementia (HR = 0.57, 95% CI 0.37–0.88, *p* = 0.0114) compared to untreated hypertensive participants (*n* = 420). When this analysis was repeated including a variable indicating suboptimally treated blood pressure (> 140 mmHg systolic or >90 mmHg diastolic), the effect of antihypertensive medication was no longer statistically significant (HR = 0.65, 95% CI 0.32–1.30, *p* = 0.2217).

**Conclusions:**

Control of blood pressure in older adult African American patients with hypertension is a key intervention for preventing dementia, with similar benefits from most of the commonly available antihypertensive medications.

**Electronic supplementary material:**

The online version of this article (10.1007/s11606-017-4281-x) contains supplementary material, which is available to authorized users.

## INTRODUCTION

Hypertension is one of the most common chronic diseases in older adults, and its prevalence is particularly high in African Americans. In the United States, compared to adults aged 18–39 years, the prevalence of hypertension in adults older than 60 years is more than eight times as great; among all adults, the prevalence is 41% for African Americans and 28% for whites.^1^^,^ 2 Studies showed that most older adults were aware of their hypertension (83%) and receive treatment (76%), but only 52% of hypertensive adults had controlled blood pressure; these results were similar for African Americans and whites.^1^ As hypertension is associated with an increased risk for dementia, one explanation for the greater risk of dementia in African Americans versus whites may be their higher rates of hypertension.^3^^–^7 This risk is especially salient given that dementia in African Americans is of mixed pathology, often involving vascular factors.^8^^–^11 Importantly, the onset of dementia results in an inexorable decline in cognitive function and long-term disability.

Effective control of hypertension with antihypertensive pharmacotherapy is an important intervention for mitigating the deleterious effects of high blood pressure leading to dementia.^12^ In a previous work, we determined that antihypertensive medications preserved cognitive function in a community-based cohort of African Americans in Indianapolis, Indiana.^13^ The odds ratio of incident cognitive impairment was 0.62 (95% CI 0.45–0.84) in subjects prescribed antihypertensives compared to those who were not. However, our study did not assess the onset of dementia, and blood pressure measurements were not sufficient to assess the effects of blood pressure and antihypertensive prescribing on the onset of dementia. The original cohort, established in 1992, was supplemented with a larger number of older adult African Americans and more complete assessment of blood pressure. In the present study, therefore, we aimed to assess the longitudinal effects of antihypertensive medications and blood pressure on the onset of dementia in a cohort of non-Hispanic African Americans with a diagnosis of hypertension spanning up to 24 years.

## METHODS

### Study Population

The study population consisted of the African American participants in the Indianapolis Ibadan Dementia Project (IIDP).^14^ The original study set out to compare cohorts from the United States (the Indianapolis cohort) and continental Africa (the Ibadan, Nigeria cohort). Because we wished to focus on the effects of antihypertensive medications commonly used in Western medicine, the Indianapolis cohort was the focus of the present study. We recruited community-dwelling participants 65 years of age or older at two time points. During the initial recruitment in 1992, 2212 African Americans aged 65 or older living in Indianapolis were enrolled. In 2001, the project enrolled 1893 additional African Americans who were 70 years of age or older. All participants agreed to undergo regular follow-up cognitive assessment and clinical evaluations by psychiatrists and neuropsychologists. Details on the original and enrichment cohorts are described elsewhere.^15^^,^ 16 The study followed a two-stage design, with a screening evaluation every 2 to 3 years, followed by a more comprehensive home-based clinical evaluation for those participants with a positive screen. Dementia was diagnosed using criteria from both the Diagnostic and Statistical Manual of Mental Disorders, Revised Third Edition (DSM-III-R),^17^ and the International Classification of Diseases, 10th Revision (ICD-10).^18^^,^^19^^–^21

### Electronic Medical Records

Electronic medical records were obtained from the Indiana Network for Patient Care (INPC) after appropriate approvals from the INPC management board.^22^ The INPC is a regional health information exchange that integrates clinical information from the major health care systems in Indiana in support of medical care, thereby reducing missing data when patients visit more than one health system for their care. These health care systems provide INPC with laboratory results, inpatient and outpatient encounter data, coded diagnoses and procedures, and digital medication records, among many other data elements. ICD-9 codes for relevant comorbidities were also obtained. Of the 4105 participants enrolled in IIDP, 122 were excluded due to prevalent dementia. Of the remaining 3982 IIDP participants without dementia, 3675 individuals were identified within Eskenazi Health, the leading provider of health care to more than one million people in Marion County (encompassing greater Indianapolis) and adjacent counties.^23^ Of these 3675 individuals, 1847 (50%) had at least three visits to primary care physicians in Eskenazi Health, with only 1400 having any prescription record during the study time frame. Our study population ultimately comprised 1236 subjects identified with a history of hypertension from either self-report from the study data or data from the electronic medical records.

### Study Endpoint

For each individual included in this analysis, baseline was defined as the date of enrollment in the study. Study endpoint was defined as the date of a dementia diagnosis for participants with incident dementia or the date of last IIDP evaluation (censored).

### Antihypertensive Medications

Prescription records for any antihypertensive medication during the period from each individual’s enrollment in the study were used to categorize overall antihypertensive medication use and subclass medication use. Prescription data were derived from Eskenazi Health prescription records and monthly updates incorporated into the INPC from Surescripts, which covers 95% of US pharmacies.^24^ Participants prescribed antihypertensive medications were grouped into classes of drugs used, as follows: alpha-beta blockers, angiotensin-converting enzyme (ACE) inhibitors, angiotensin II receptor blockers, beta-adrenergic blockers, calcium channel blockers, centrally acting adrenergic agonists, and diuretics (thiazide and thiazide-like, loop, and combination). Several individual subcategories including aldosterone blockers, alpha blockers, peripherally acting antiadrenergic agents, and vasodilators were rarely prescribed, which precluded our ability to ascertain valid odds ratio estimates, but these drugs were included in the overall estimates of antihypertensive medication effect.

### Blood Pressure Measures

Blood pressure was measured for all consenting participants, starting from the second follow-up evaluation in 1997—i.e. year 5 after baseline for those enrolled in the 1992 cohort—and at all evaluations for the 2001 cohort. Three measurements of seated systolic and diastolic blood pressure were taken from the left arm by trained interviewers using Omron digital input/output units (Omron Healthcare Inc., Bannockburn, IL) at approximately 15-min intervals. The average of the three measurements was recorded and used in all analyses.

### APOE Genotype

DNA was extracted from blood spots collected on filter paper and from fresh blood, using standard procedures, from participants who consented to the procedure in 2001 or before. APOE genotypes were determined by HhaI digestion of amplified products.^25^

### Statistical Analyses

We classified hypertensive participants into two mutually exclusive groups: those who were prescribed antihypertensive therapy and those who were not. Individuals were considered as having a history of hypertension based on participant self-reports or reports of family members during IIDP evaluations, or based on ICD-9 codes for the diagnosis of hypertension (401.0, 401.1, and 401.9) in the electronic medical record. For analyses of antihypertensive subclasses, we divided the group using any antihypertensive medication into two groups: those using a particular medication subclass and those using more than one subclass of antihypertensive medication.

We used *t* tests for continuous variables and chi-square tests for categorical variables to compare baseline characteristics between participants with incident dementia and those without dementia. Analysis of variance was used for continuous variables and Fisher’s exact tests for frequency variables to compare participant characteristics, comorbid conditions, and blood pressure measurements among participants taking any antihypertensive medication, various subclasses, or no antihypertensive medication. We plotted Kaplan–Meier curves to assess the temporal relationship of incident dementia in participants prescribed and not prescribed antihypertensive medications, and used the log-rank test to compare the temporal distributions of the curves. Cox proportional hazards regression models were used to test the association of overall and subclass antihypertensive medications with time to dementia. Covariates included baseline age, gender, education, and comorbid medical conditions. Blood pressure measures collected from repeated evaluations were included in the Cox models as an indicator variable for suboptimal blood pressure control, defined as all blood pressure measurements from a subject >140 mmHg systolic or >90 mmHg diastolic.

We also conducted sensitivity analyses using propensity scores to control for potential biases due to treatment indication. A logistic model was used to derive predictive probability (propensity scores) for an antihypertensive medication using baseline variables. Cox models for time to dementia were stratified by quintiles of propensity.

### Ethics

Informed consent was obtained from all study participants, and the study was approved by the Indiana University–Purdue University institutional review board (IRB).

## RESULTS

Of the 1236 hypertensive participants without dementia at baseline, 114 (9%) developed incident dementia during follow-up evaluations. In Table 1, we include comparisons of participant characteristics, medical conditions, and blood pressure measures between the incident dementia and non-dementia groups. Individuals with incident dementia were significantly older at baseline, with fewer years of education, were more likely to be carriers of the APOE ε4 allele, and had higher initial systolic blood pressure than those who were not diagnosed with dementia. However, there were no statistically significant differences between the groups with respect to the remaining variables compared in Table 1.

**Table 1:** Characteristics of Participants with Hypertension at Baseline and during Follow-Up in the Indianapolis Ibadan Dementia Project (IIDP) by Dementia Status

	With dementia		Without dementia		
Characteristic	*N*	Mean ± SD or no. (%)	*N*	Mean ± SD or no. (%)	*P* value
**Baseline:**					
Mean age (SD), years	114	76.9 ± 6.9	1122	74.7 ± 6.0	<0.001
Male, no. (%)	114	26 (22.8%)	1122	304 (26.5%)	0.57
Years of education (SD)	114	8.8 ± 3.3	1120	10.1 ± 2.9	<0.001
Stroke, no. (%)	114	7 (6.1%)	1122	63 (5.6%)	0.83
Heart failure, no. (%)	114	32 (28.1%)	1122	300 (26.7%)	0.74
Chronic renal disease, no. (%)	114	10 (8.8%)	1122	72 (6.4%)	0.32
Coronary artery disease, no. (%)	114	39 (34.2%)	1122	319 (28.4%)	0.20
Diabetes - type 1 or 2, no. (%)	114	41 (36.0%)	1122	414 (36.9%)	0.92
Atrial fibrillation, no. (%)	114	10 (8.8%)	1122	114 (10.2%)	0.74
Transient ischemic attack, no. (%)	114	10 (8.8%)	1122	67 (6.0%)	0.22
Chronic obstructive pulmonary disease, no. (%)	114	5 (4.4%)	1122	91 (8.1%)	0.20
Cancer, no. (%)	114	41 (36.0%)	1122	481 (42.9%)	0.16
ApoE ε4 carriers, no. (%)	82	38 (46.3%)	625	216 (34.6%)	0.05
Baseline systolic blood pressure (SD), mmHg	74	152.5 ± 26.0	766	145.8 ± 22.3	0.02
Baseline diastolic blood pressure (SD), mmHg	74	78.1 ± 14.9	766	77.8 ± 12.2	0.88
**Follow-Up:**					
Mean systolic blood pressure (SD), mmHg*	74	150.0 ± 21.5	766	145.3 ± 20.0	0.05
Mean diastolic blood pressure(SD), mmHg*	74	76.6 ± 12.0	766	76.1 ± 10.5	0.72
Blood pressure suboptimally treated, no. (%)^†^	74	35 (47.3%)	766	304 (39.7%)	0.22
Died, no. (%)^‡^	114	66 (57.9%)	1122	511 (45.5%)	0.01
Mean age at endpoint (SD), years	114	84.8 ± 6.8	1122	80.8 ± 6.5	<0.001

*Average blood pressure measure per person from repeated blood pressure measurements

^†^Defined as all blood pressure measurements >140 mmHg systolic or >90 mmHg diastolic pressure during follow-up

^‡^Died between reaching study endpoint and the end of study (2/25/2010)

**Figure 1:**
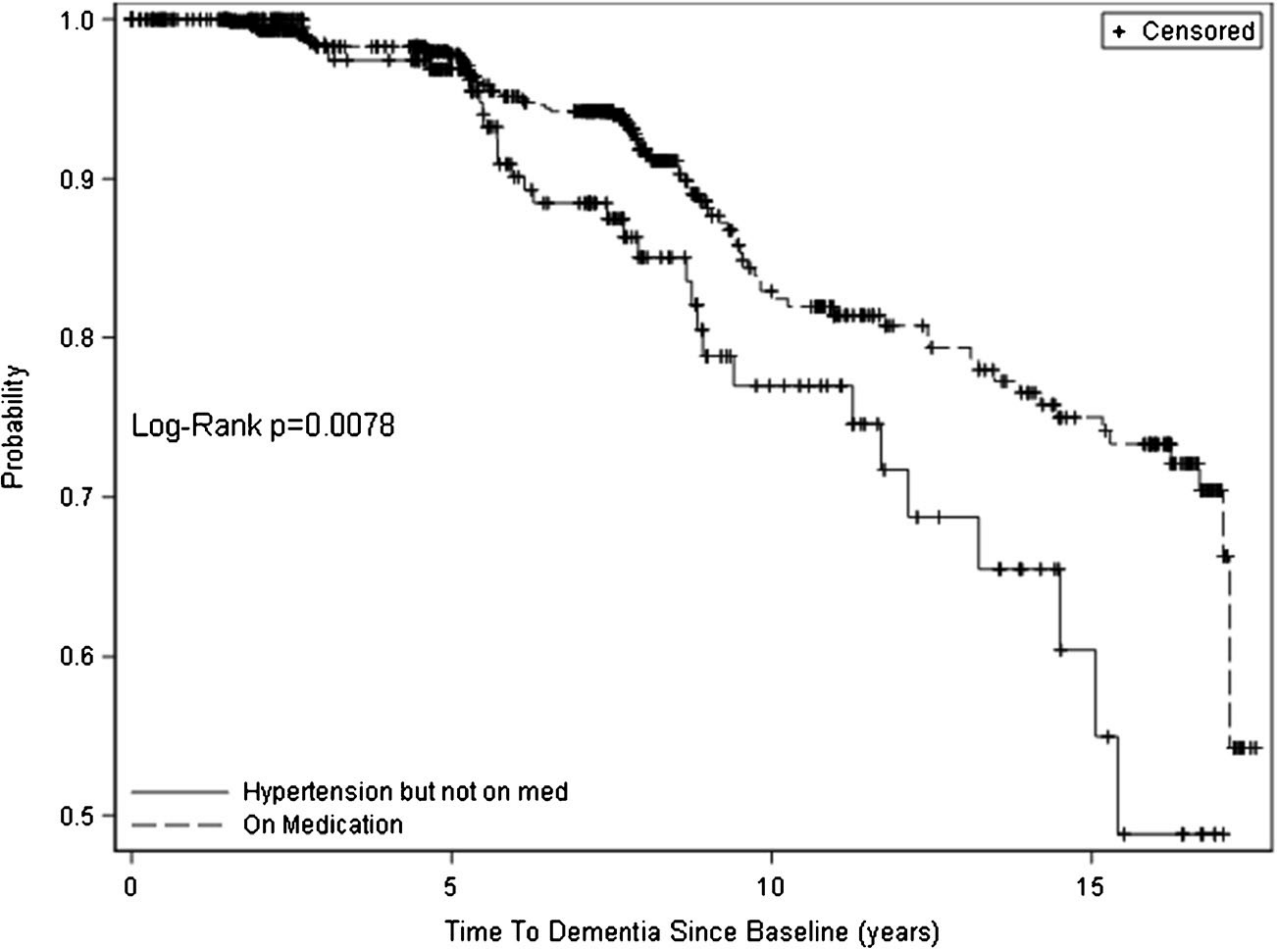
Kaplan–Meier curves of the probability of dementia over time for participants prescribed an antihypertensive medication (solid line) vs. those not prescribed an antihypertensive medication (dashed line).

In Table 2, we compare the same set of participant characteristics between those treated with antihypertensive medication and those not treated. Individuals prescribed medications had significantly higher proportions of comorbid conditions than participants with untreated hypertension. Despite the greater burden of cardiovascular and other comorbid diagnoses in those treated with antihypertensives, mortality was higher in the untreated group, possibly from events associated with elevated blood pressure. The Kaplan–Meier plot (see Fig. 1) shows the temporal distributions of incident dementia for participants prescribed antihypertensive medication and those who were untreated. Inspection of the curves shows separation at about 5 years that continues through the course of 15 years (*p* = 0.0078).

**Table 2:** Baseline Characteristics of Participants with Hypertension in the Indianapolis Ibadan Dementia Project (IIDP) by Antihypertensive Treatment Status

Variable	Not prescribed antihypertensive		Prescribed antihypertensive		*P* value
	*N*	No. (%)	*N*	No. (%)	
Stroke	420	19 (4.5%)	816	51 (6.3%)	0.24
Heart failure	420	61 (14.5%)	816	271 (33.2%)	<0.001
Chronic renal disease	420	17 (4.0%)	816	65 (8.0%)	0.007
Coronary artery disease	420	92 (21.9%)	816	266 (32.6%)	<0.001
Diabetes - type 1 or 2	420	113 (26.9%)	816	342 (41.9%)	<0.001
Atrial fibrillation	420	24 (5.7%)	816	100 (12.3%)	<0.001
Transient ischemic attack	420	13 (3.1%)	816	64 (7.8%)	<0.001
Chronic obstructive pulmonary disease	420	25 (6.0%)	816	71 (8.7%)	0.09
Cancer	420	114 (27.1%)	816	408 (50.0%)	<0.001
ApoE ε4 carriers	190	69 (36.3%)	517	185 (35.8%)	0.93
Blood pressure suboptimally treated	162	84 (51.9%)	678	255 (37.6%)	0.001
Mean years of follow-up	420	3.9 ± 4.3	816	7.5 ± 4.4	<0.001
Died*	420	214 (51.0%)	816	363 (44.5%)	0.035

*Died between reaching study endpoint and the end of study (2/25/2010)

In Table 3, we present results from Cox models for any antihypertensive treatment and time to dementia while adjusting for demographic variables and comorbid medical conditions. Individuals prescribed any antihypertensive medication were found to have a significantly reduced risk of dementia (HR = 0.57, 95% CI 0.37–0.88, *p* = 0.0114) compared to untreated hypertensive participants. None of the medical conditions considered were significantly associated with dementia risk. A somewhat unusual finding was that the risk of dementia was lower in participants with a diagnosis of cancer (HR = 0.61, 95% CI 0.41–0.90, *p* = 0.0133). In a subset of patients who consented to blood sample collection (*n* = 707), we further adjusted the model by APOE ε4 carrier status. Individuals prescribed any antihypertensive medication still showed a lower risk of dementia (HR = 0.65, 95% CI 0.38–1.12, *p* = 0.12) than untreated hypertensive participants, but the result was no longer significant. When this analysis was repeated including a variable indicating suboptimally treated blood pressure (> 140 mmHg systolic or >90 mmHg diastolic), the effect of antihypertensive medication was no longer statistically significant (HR = 0.65, 95% CI 0.32–1.30, *p* = 0.2217; see Table 3). This latter effect suggests that once we included the presence of suboptimal treatment in the Cox model, the association between antihypertensive medication and incident dementia risk was no longer significant. A propensity-adjusted Cox model using variables in Table 3 also revealed a significantly reduced risk of dementia (HR = 0.33, 95% CI 0.14–0.80, *p* = 0.0135).

**Table 3:** Results of Cox Proportional Hazards Models of Time to Dementia for Older Adult Patients with Hypertension by Any Antihypertensive Medication Usage, With and Without Adjusting for Blood Pressure Control*

Variable	Models without adjusting for ApoE ε4			Models adjusting for ApoE ε4		
	Models without adjusting for blood pressure control (*n* = 1234 and 707)					
	HR	95% CI	*p* value	HR	95% CI	*p* value
Prescribed any antihypertensive medication	0.57	0.37–0.88	0.01	0.65	0.38–1.12	0.12
Not prescribed antihypertensive medication	Ref			Ref		
Age at baseline	1.11	1.08–1.15	<0.001	1.12	1.08–1.16	<0.001
Years of education	0.89	0.84–0.95	<0.001	0.88	0.82–0.95	<0.001
Male	0.96	0.61–1.53	0.87	0.85	0.46–1.55	0.59
Female						
Coronary artery disease	1.03	0.66–1.60	0.90	0.85	0.51–1.42	0.53
Chronic renal disease	0.94	0.48–1.83	0.85	1.32	0.63–2.77	0.46
Heart failure	1.10	0.67–1.80	0.72	1.13	0.63–2.03	0.69
Transient ischemic attack	1.14	0.57–2.27	0.71	1.21	0.55–2.70	0.64
Stroke	1.56	0.70–3.51	0.28	1.53	0.62–3.76	0.35
Atrial fibrillation	0.69	0.34–1.39	0.30	0.83	0.39–1.78	0.63
Diabetes - type 1 or 2	0.92	0.62–1.38	0.69	0.85	0.51–1.40	0.52
Chronic obstructive pulmonary disease	0.45	0.18–1.15	0.10	0.54	0.21–1.44	0.22
Cancer	0.61	0.41–0.90	0.01	0.55	0.34–0.87	0.011
ApoE ε4 carriers	N/A	N/A	N/A	2.07	1.30–3.29	0.002
	Models adjusting for blood pressure control (*n* = 838 and 560)					
Prescribed any antihypertensive medication	0.65	0.32–1.30	0.22	0.96	0.38–2.44	0.93
Not prescribed antihypertensive medication	Ref			Ref		
Age at baseline	1.13	1.08–1.17	<0.001	1.15	1.10–1.20	<0.001
Years of education	0.88	0.81–0.95	0.002	0.85	0.77–0.95	0.003
Male	0.96	0.54–1.73	0.90	1.06	0.49–2.29	0.87
Female						
Blood pressure suboptimally treated	1.78	1.11–2.86	0.02	2.19	1.22–3.94	0.009
Coronary artery disease	1.15	0.66–2.02	0.62	0.99	0.51–1.92	0.98
Chronic renal disease	1.01	0.45–2.26	0.98	1.37	0.55–3.40	0.50
Heart failure	1.23	0.65–2.30	0.52	1.09	0.52–2.28	0.82
Transient ischemic attack	1.34	0.63–2.89	0.45	1.44	0.58–3.55	0.43
Stroke	1.15	0.38–3.48	0.81	0.86	0.23–3.16	0.82
Atrial fibrillation	0.60	0.26–1.40	0.24	0.74	0.29–1.90	0.53
Diabetes - type 1 or 2	0.70	0.42–1.18	0.18	0.72	0.37–1.38	0.32
Chronic obstructive pulmonary disease	0.40	0.12–1.33	0.14	0.51	0.15–1.77	0.29
Cancer	0.76	0.47–1.24	0.27	0.72	0.40–1.28	0.26
ApoE ε4 carriers	N/A	N/A	N/A	2.11	1.15–3.88	0.017

*HR = hazard ratio; 95% CI = 95% confidence interval

Online Supplementary Table 1 contains the results of the multiple Cox proportional hazards regression model for antihypertensive subcategories. For each antihypertensive type, the overall effect of the drug was to prevent or delay progression to dementia, with the exception of alpha-beta blockers and central adrenergic agonists. When these analyses were repeated including a variable indicating suboptimally treated blood pressure, the effect of each antihypertensive medication was no longer statistically significant, with the exception of loop diuretics (HR = 0.36, 95% CI 0.15–0.87, *p* = 0.0233; see Table S2 in the Online Supplementary Appendix). Thus, with the exception of loop diuretics, poorly controlled blood pressure—i.e. ineffective treatment—diminished the cognitive preservation effects of the drug.

## DISCUSSION

We studied the effects of antihypertensive treatment on the risk of dementia over a period of up to 24 years in a cohort of 1262 African Americans with hypertension in the IIDP. We found that, compared to untreated participants, those prescribed antihypertensives had a 43% lower risk of dementia (HR = 0.57, 95% CI 0.37–0.88, *p* = 0.0114). These effects were consistent for commonly used antihypertensive medications, an important consideration when prescribing for optimal medication effectiveness and cost considerations such as brand name versus generic. When taking into account the effect of poorly controlled hypertension—i.e. blood pressure consistently >140 mmHg systolic or >90 mmHg diastolic—the risk of dementia was almost twice as great (HR = 1.78, 95% CI 1.11–2.86, *p* = 0.0166), and the effect of antihypertensive medication decreased to a 35% reduction in dementia risk, which was no longer statistically significant (HR = 0.65, 95% CI 0.32–1.30, *p* = 0.12217). We interpret these findings as suggesting that the risk of dementia is largely due to blood pressure control, regardless of the antihypertensive drug class(es) prescribed.

The results of the present study are consistent with our earlier findings in the IIDP cohort investigating the effect of antihypertensive medications on incident cognitive impairment. In the previous study, we found that antihypertensive medications reduced the odds of incident cognitive impairment in the IIDP African American cohort by 38% (OR = 0.62, 95% CI 0.45–0.84).^13^ However, we did not have sufficient blood pressure data to determine the effect of blood pressure for the various antihypertensive medications that had been prescribed.

Our present results are also consistent with studies of African Americans by colleagues from the Atherosclerosis Risk in Communities (ARIC) study, which assessed dementia and cognitive decline in both white and African American participants.^3^^,^ 4 However, the data comparing the risk of dementia and cognitive decline in longitudinal cohorts is conflicting. Alonso and colleagues found a higher risk of hospitalization with dementia in African Americans in the ARIC study, which included 2619 African Americans (age-adjusted HR = 2.5, 95% CI 1.9–3.3) and 8532 whites (age-adjusted HR = 1.0, 95% CI 0.7–1.3), presumably due to a greater overall burden of cardiovascular disorders (hypercholesterolemia, smoking, diabetes, and hypertension).^3^ However, dementia hazard ratios for hypertension were similar between African Americans (1.7) and whites (1.6). More recently, Gottesman and colleagues determined changes in cognitive scores in ARIC participants over the course of 20 years, and found a significant decline in cognitive scores in participants with hypertension that was greater in whites than African Americans and greater in those not prescribed antihypertensives versus those who received antihypertensives.^4^ In contrast, Katz and colleagues found that race was a significant risk factor for mild cognitive impairment but not for dementia in participants 70 years of age and older in the community-based Einstein Aging Study, after controlling for sex and education.^26^

Some investigators have suggested that certain classes of antihypertensive drugs may have greater neuroprotective effects than others.^27^^–^29 However, we found little evidence (with the exception of loop diuretics) for such effects across commonly used antihypertensive medications. It is possible that the greater natriuretic potency of loop diuretics contributes further to reducing blood pressure. A recent meta-analysis of risk factors for Alzheimer’s disease suggests a general protective effect for various drug classes including antihypertensives, estrogen, nonsteroidal anti-inflammatory drugs, and statins.^7^ Collectively, these drugs could play some role in protecting against a variety of cardiovascular insults that might otherwise contribute to cognitive changes and dementia in susceptible persons such as those with mixed-pathology dementia.^8^

Differences among antihypertensive drugs may also reflect patient adherence. Poon and colleagues presented data suggesting that, in addition to a higher prevalence of hypertension in African Americans than whites, poorer adherence to antihypertensive medication may contribute to risk of dementia.^30^^,^ 31 Ritchey and colleagues also found poorer adherence in African Americans in a study of Medicare Part D beneficiaries.^32^ While it is possible that adherence to and tolerance of antihypertensives may be better for some drug classes than others, our results suggest that the key to preventing dementia is blood pressure control. Patients who progressed to dementia may have had more erratic medication adherence patterns, but no participants had dementia at baseline, and medication use was assessed within the period before diagnosis of dementia. Placebo effects cannot be ruled out in our study.

The lower risk of dementia found for participants with cancer (HR = 0.61) has been reported with similar effect sizes in other dementia cohorts (HR = 0.65)^33^ and in a recent meta-analysis of cohort studies (HR = 0.63).^34^ This reduced risk in individuals with cancer could represent a survivor effect or diagnostic bias, or it could be explained by a protective effect of cancer chemotherapy, but is a finding that is beyond the scope of the present study.

Our study has several limitations. Unfortunately, we do not have a sufficient number of participants with pure vascular dementia for a separate model. However, we conducted additional analyses of stroke and non-Alzheimer's dementia, and found that the overall effect size for the use of any antihypertensive medication was unaffected. We also focused our results on treated versus untreated hypertensive African Americans in our IIDP cohort, which resulted in a smaller cohort for study, and the effects of some of the less frequently prescribed subclasses of antihypertensives could not be reliably determined. It may seem unusual that there are patients with hypertension who are not receiving an antihypertensive medication. However, a recent National Health and Nutrition Examination Survey (NHANES) indicated that 24% of patients with hypertension were not receiving an antihypertensive agent.^1^ Others have had similar findings, including the Atherosclerosis Risk in Communities Neurocognitive Study, in which only 78.7% of white and 82.2% of African American participants with hypertension were being treated with antihypertensives.^4^ Presumably, patients who were not receiving medical treatment had milder hypertension managed with lifestyle modifications such as weight loss or low-sodium diets.^12^ In addition, some of the subclasses, such as ACE inhibitors and beta blockers, could have been used to treat other disorders in addition to hypertension. Nonetheless, the effects of these medications were generally consistent but sensitive to the inclusion of an indicator variable for poorly controlled blood pressure. Furthermore, our study data now span up to 24 years, with consistently rigorous screening and neurological assessment of cognitive impairment and dementia. Another limitation, which is faced by many cohort studies in the elderly population, is the possibility of differential dropout due to death, which may threaten the validity of the random censoring assumption required for the modeling approach. Additional models using semi-competing risk models may be required to adequately determine whether our results would change after adjusting for death.

While the rates of dementia are declining in the United States despite increases in cardiovascular comorbidities, African Americans have a disproportionate share of cardiovascular risk factors for its development.^11^ Control of hypertension is a key preventive intervention for reducing the risk of dementia as well as other cardiovascular insults such as myocardial infarction and stroke. Studies published to date have predominately focused on hypertension at mid-life and dementia risk, whereas our cohort participants were elderly at the time of study enrollment. Thus the results of the present study extend these earlier observations by investigating the association between hypertension later in life and risk of dementia, and the importance of reducing blood pressure with effective pharmacotherapy to prevent the onset of dementia even in older adults with hypertension.

## CONCLUSION

Control of blood pressure in older adult African American patients with hypertension is a key intervention for preventing dementia, with similar benefits from most of the commonly available antihypertensive medications.

## Electronic supplementary material

ESM 1 (DOCX 22 kb)
