# Supplementary material for: Antihypertensive Medication and Dementia Risk in Older Adult African Americans with Hypertension: A Prospective Cohort Study
Source: J Gen Intern Med. 2018 Jan 12;33(4):455–62. doi: 10.1007/s11606-017-4281-x (PMC5880772; doi:10.1007/s11606-017-4281-x)
Supplement: Supplementary file 1 — (DOCX 22 kb) [file 11606_2017_4281_MOESM1_ESM.docx]

**Supplementary Table S1. Multiple Cox Proportional Hazards Models of Time to Dementia for Older Adult Patients with Hypertension by Antihypertensive Medication Subclass Status***

| *Antihypertensive Status* | *HR* | *95% CI* | *p-value* |
| --- | --- | --- | --- |
| ACE inhibitor: Overall effect | . |  | 0.03 |
| Taking an ACE inhibitor | 0.62 | 0.38-0.99 | 0.05 |
| Taking antihypertensive but not an ACE inhibitor | 0.52 | 0.31-0.87 | 0.17 |
| Not prescribed antihypertensive medication | . |  | . |
|  | . |  | . |
| Angiotensin II receptor blocker: Overall effect |  |  | 0.02 |
| Taking an angiotensin II receptor blocker | 0.34 | 0.15-0.77 | 0.01 |
| Taking antihypertensive by not an angiotensin II receptor blocker | 0.62 | 0.40-0.95 | 0.03 |
| Not prescribed antihypertensive medication | . |  | . |
|  |  |  |  |
| Beta-blocker: Overall effect | . |  | 0.009 |
| Taking a beta-blocker | 0.43 | 0.26-0.74 | 0.002 |
| Taking antihypertensive but not a beta-blocker | 0.69 | 0.43-1.10 | 0.12 |
| Not prescribed antihypertensive medication | . |  | . |
|  |  |  |  |
| Alpha-beta-blocker: Overall effect | . |  | 0.03 |
| Taking an alpha-beta-blocker | 0.83 | 0.28-2.46 | 0.73 |
| Taking antihypertensive but not an alpha-beta-blocker | 0.57 | 0.37-0.87 | 0.01 |
| Not prescribed antihypertensive medication | . |  | . |
|  |  |  |  |
| Cardioselective beta-blocker: Overall effect | . |  | 0.004 |
| Taking a cardioselective beta-blocker | 0.40 | 0.23-0.69 | 0.001 |
| Taking antihypertensive but not a cardioselective beta-blocker | 0.71 | 0.45-1.13 | 0.15 |
| Not prescribed antihypertensive medication | . |  | . |
|  |  |  |  |
| Calcium channel blocker: Overall effect | . |  | 0.04 |
| Taking a calcium channel blocker | 0.59 | 0.37-0.95 | 0.03 |
| Taking antihypertensive but not a calcium channel blocker | 0.54 | 0.33-0.91 | 0.02 |
| Not prescribed antihypertensive medication | . |  | . |
|  |  |  |  |
| Diuretic: Overall effect | . |  | 0.03 |
| Taking Any type of diuretic | 0.54 | 0.34-0.84 | 0.01 |
| Taking antihypertensive but not a diuretic | 0.71 | 0.39-1.27 | 0.25 |
| Not prescribed antihypertensive medication | . |  | . |
|  |  |  |  |
| Loop diuretics: Overall effect | . |  | 0.02 |
| Taking a loop diuretic | 0.43 | 0.24-0.78 | 0.005 |
| Taking antihypertensive but not a loop diuretic | 0.64 | 0.41-0.99 | 0.05 |
| Not prescribed antihypertensive medication | . |  | . |
| Taking a thiazide diuretic | 0.63 | 0.39-1.02 | 0.06 |

| *Antihypertensive Status* | *HR* | *95% CI* | *p-value* |
| --- | --- | --- | --- |
| Thiazide diuretic: Overall effect | . |  | 0.03 |
| Taking a thiazide diuretic | 0.63 | 0.39-1.02 | 0.06 |
| Taking antihypertensive but not a thiazide diuretic | 0.52 | 0.31-0.85 | 0.008 |
| Not prescribed antihypertensive medication | . |  | . |
|  |  |  |  |
| Diuretic combination: Overall effect | . |  | 0.04 |
| Taking a diuretic combination | 0.54 | 0.29-0.99 | 0.05 |
| Taking antihypertensive but not a diuretic combination | 0.58 | 0.37-0.91 | 0.02 |
| Not prescribed antihypertensive medication | . |  | . |
|  |  |  |  |
| Central adrenergic agonist: Overall effect | . |  | 0.04 |
| Taking a central adrenergic agonist | 0.56 | 0.25-1.27 | 0.16 |
| Taking an antihypertensive but not a central adrenergic agonist | 0.57 | 0.37-0.89 | 0.01 |
| Not prescribed antihypertensive medication | . |  | . |

*Adjusted for age, gender, years of education, and relevant comorbidities; HR=hazard ratio; 95% CI=95% confidence interval

[Supplemental Appendix]

**Supplementary Table S2. Multiple Cox Proportional Hazards Models of Time to Dementia for Older Adult Patients with Hypertension by Antihypertensive Subclass Adjusting for Blood Pressure Control***

|  |  |  |  |
| --- | --- | --- | --- |

| *Variable* | *HR* | *95% CI* | *p-value* |
| --- | --- | --- | --- |
| ACE inhibitor: Overall effect | . |  | 0.39 |
| Taking an ACE inhibitor | 0.71 | 0.33-1.49 | 0.36 |
| Taking antihypertensive but not an ACE inhibitor | 0.59 | 0.27-1.26 | 0.17 |
| Not prescribed antihypertensive medication | . |  | . |
| Blood pressure suboptimally treated † | 1.79 | 1.12-2.88 | 0.02 |
|  |  |  |  |
| Angiotensin II receptor blocker: Overall effect |  |  | 0.27 |
| Taking an angiotensin II receptor blocker | 0.44 | 0.16-1.20 | 0.11 |
| Taking antihypertensive by not an angiotensin II receptor blocker | 0.67 | 0.33-1.36 | 0.27 |
| Not prescribed antihypertensive medication | . |  | . |
| Blood pressure suboptimally treated † | 1.83 | 1.14-2.95 | 0.013 |
|  |  |  |  |
| Beta-blocker: Overall effect | . |  | 0.22 |
| Taking a beta-blocker | 0.52 | 0.24-1.14 | 0.10 |
| Taking antihypertensive but not a beta-blocker | 0.74 | 0.36-1.54 | 0.43 |
| Not prescribed antihypertensive medication | . |  | . |
| Blood pressure suboptimally treated † | 1.77 | 1.10-2.84 | 0.02 |
|  |  |  |  |
| Alpha-beta-blocker: Overall effect | . |  | 0.47 |
| Taking an alpha-beta-blocker | 0.61 | 0.12-2.96 | 0.54 |
| Taking antihypertensive but not an alpha-beta-blocker | 0.65 | 0.32-1.30 | 0.22 |
| Not prescribed antihypertensive medication | . |  | . |
| Blood pressure suboptimally treated † | 1.78 | 1.11-2.86 | 0.02 |
|  |  |  |  |
| Cardioselective beta-blocker: Overall effect | . |  | 0.19 |
| Taking a cardioselective beta-blocker | 0.50 | 0.23-1.11 | 0.09 |
| Taking antihypertensive but not a cardioselective beta-blocker | 0.75 | 0.36-1.55 | 0.44 |
| Not prescribed antihypertensive medication | . |  | . |
| Blood pressure suboptimally treated † | 1.76 | 1.10-2.83 | 0.02 |
|  |  |  |  |
| Calcium channel blocker: Overall effect | . |  | 0.46 |
| Taking a calcium channel blocker | 0.66 | 0.32-1.38 | 0.27 |
| Taking antihypertensive but not a calcium channel blocker | 0.62 | 0.29-1.35 | 0.23 |
| Not prescribed antihypertensive medication | . |  | . |
| Blood pressure suboptimally treated † | 1.77 | 1.10-2.85 | 0.02 |
|  |  |  |  |
|  |  |  |  |
| *Variable* | *HR* | *95% CI* | *p-value* |
| Diuretic: Overall effect | . |  | 0.47 |
| Taking Any type of diuretic | 0.65 | 0.31-1.33 | 0.23 |
| Taking antihypertensive but not a diuretic | 0.65 | 0.27-1.55 | 0.33 |
| Not prescribed antihypertensive medication | . |  | . |
| Blood pressure suboptimally treated † | 1.78 | 1.11-2.86 | 0.02 |
|  |  |  |  |
| Loop diuretics: Overall effect | . |  | 0.05 |
| Taking a loop diuretic | 0.36 | 0.15-0.87 | 0.02 |
| Taking antihypertensive but not a loop diuretic | 0.75 | 0.37-1.51 | 0.42 |
| Not prescribed antihypertensive medication | . |  | . |
| Blood pressure suboptimally treated † | 1.93 | 1.20-3.13 | 0.01 |
|  |  |  |  |
| Thiazide diuretic: Overall effect | . |  | 0.07 |
| Taking a thiazide diuretic | 0.82 | 0.40-1.70 | 0.60 |
| Taking antihypertensive but not a thiazide diuretic | 0.47 | 0.22-1.03 | 0.06 |
| Not prescribed antihypertensive medication | . |  | . |
| Blood pressure suboptimally treated † | 1.74 | 1.08-2.80 | 0.02 |
|  |  |  |  |
| Diuretic combination: Overall effect | . |  | 0.47 |
| Taking a diuretic combination | 0.66 | 0.28-1.56 | 0.35 |
| Taking antihypertensive but not a diuretic combination | 0.64 | 0.32-1.31 | 0.22 |
| Not prescribed antihypertensive medication | . |  | . |
| Blood pressure suboptimally treated † | 1.78 | 1.11-2.86 | 0.02 |
|  |  |  |  |
| Central adrenergic agonist: Overall effect | . |  | 0.20 |
| Taking a central adrenergic agonist | 0.32 | 0.09-1.12 | 0.08 |
| Taking an antihypertensive but not a central adrenergic agonist | 0.66 | 0.33-1.33 | 0.25 |
| Not prescribed antihypertensive medication | . |  | . |
| Blood pressure suboptimally treated † | 1.88 | 1.17-3.03 | 0.01 |
|  |  |  |  |

*Adjusted for age, gender, years of education, and relevant comorbidities. HR=hazard ratio; 95% CI = 95% confidence interval.

† Defined as all blood pressure measures > 140 mmHg systolic or > 90 mmHg diastolic during follow-up.
